# Saving a Child's Elbow Joint: A Novel Reconstruction for a Tumour of the Distal Humerus

**DOI:** 10.1155/2015/404979

**Published:** 2015-01-08

**Authors:** Calogero Graci, Czar Louie Gaston, Robert Grimer, Lee Jeys, Korhan Ozkan

**Affiliations:** ^1^Department of Orthopedics and Traumatology, University Hospital “Agostino Gemelli”, School of Medicine, Catholic University of the Sacred Heart, Largo A. Gemelli 1, 00168 Rome, Italy; ^2^Department of Musculoskeletal Oncology, The Royal Orthopaedic Hospital, Birmingham B31 2AP, UK; ^3^Department of Orthopaedics and Traumatology, ISMU Göztepe Education and Research Hospital, Faculty of Medicine, Istanbul Medeniyet University, 34732 Istanbul, Turkey

## Abstract

Reconstruction after wide resection of a malignant bone tumor can be obtained using several techniques such as the use of prostheses, allograft, autograft, or combined procedure. We describe a 12-year-old girl with parosteal osteosarcoma of the distal right humerus treated by en bloc resection, intraoperative extracorporeal irradiation, and implantation. We inserted a nonvascularised fibular autograft through the middle of irradiated graft to obtain a greater stability. We have not recorded any complication associated with this technique such as nonunion, pathological fracture, infection, and bone necrosis and we obtained an excellent functional result. 10 years after surgery, the patient had no recurrence. Extracorporeal irradiation and reimplantation is a valid and inexpensive technique for the treatment of bone tumors when there is reasonable residual bone stock. With this procedure we have a precise fit being the patient's own bone. In this way we avoid all the problems related to the adaptation of the shape and size.

## 1. Introduction

In recent decades the treatment and prognosis of osteosarcoma have changed. Radical surgery, based on amputation, has been replaced in many cases by limb salvage surgery, with “en bloc” resection of the tumor and reconstruction by prostheses, allograft, or combined methods.

An interesting alternative reconstruction technique in the treatment of primary bone tumours is represented by resection of the bone part affected by the tumor, extracorporeal irradiation, and reimplantation. Thanks to the results obtained and presented in literature, even in the long term, this method represents in certain cases a valid technique [1, 2].

Certainly the advantages of the use of prostheses are different; they are easy to find and allow rapid mobility and weight bearing. However, the implants are not free from complications, especially in the long term. Loosening and breakage are the most frequent complications of prostheses, especially in young patients [3–5]. Even the use of allograft is linked to some problems, such as the availability of a bone bank and risk of disease transmission. In Asian countries, the allograft is avoided for religious reasons.

Use of the autograft means avoiding many of these issues. We will not have the problem of availability and risk of disease transmission and costs are considerably lower. The problem of size and shape adaptation does not exist and we also have a natural scaffold for reattachment of tendons and ligaments to original site [6]. As demonstrated in many studies, the irradiation of autograft provides for the eradication of all tumor cells [1, 2].

We describe here a case of a young girl with parosteal osteosarcoma of the distal right humerus, an unusual tumor lesion at this age and at this location. This lesion was treated by en bloc resection, intraoperative extracorporeal irradiation, and reimplantation, preserving natural joint. We inserted a nonvascularised fibular autograft through the middle of irradiated graft to provide extra stability. We have not had any complications and we obtained an excellent functional result.

We believe that this technique may provide a rewarding alternative to allografting and in some cases can replace the use of tumor implants with excellent functional and oncological results.

This technique may be applicable for tumors with a strong bone composition type like a parosteal osteosarcoma. In our opinion, this technique is not indicated for bone lytic lesions that decrease the biomechanical properties of bone. Subjecting the graft to extracorporeal irradiation and reimplantation would make the bone even weaker, both during surgery and during fixation once implanted.

## 2. Case Report

A 12-year-old woman presented with a painless swelling in the distal aspect of the right humerus of 1 month's duration. She did not report any trauma to the arm. Physical examination revealed a firm, nontender swelling deep to the triceps just above the elbow. The function of the right upper limb was normal, with a full range of movements in all joints and without distal neurovascular deficit and no regional lymphadenopathy.

**Figure 1:**
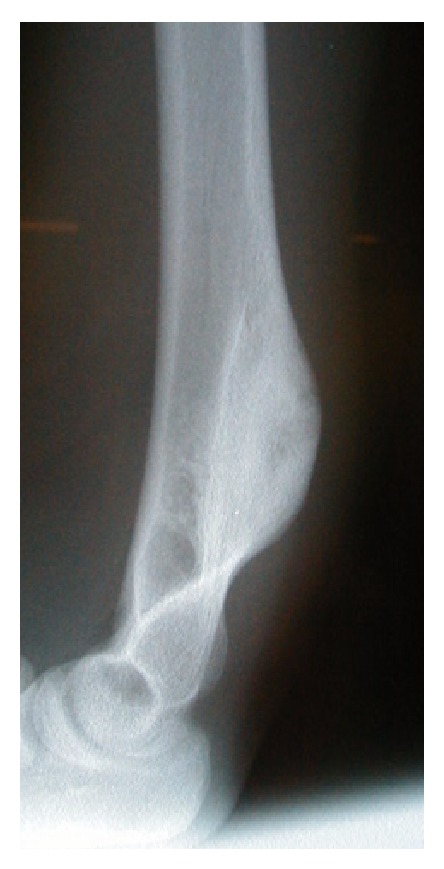
Lateral X-ray of the distal right humerus showing a dense bony protuberance arising from the distal aspect of the humerus on the posterior surface without periosteal reaction.

**Figure 2:**
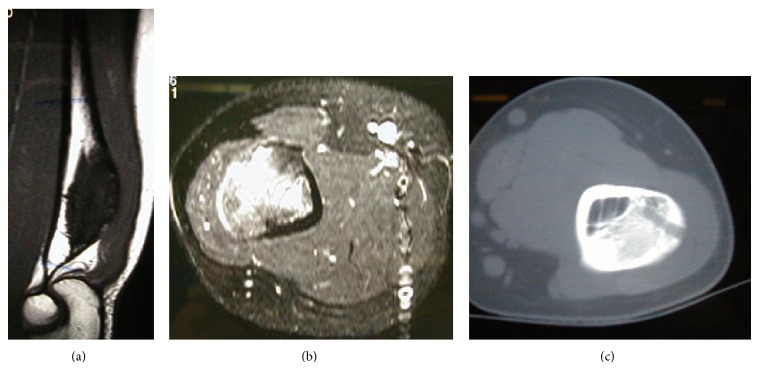
The MRI images show an exostosis arising from the posterior surface of the distal humerus. This lesion measured approximately 6 cm in its long axis terminating immediately proximally to the level of the olecranon and with involvement of the underlying medulla. You can see the sign of the biopsy (c).

Plain X-rays showed a dense sclerotic bony protuberance arising from the posterior cortex of the distal aspect of the humerus (Figure 1). The MRI showed an exostosis measuring 6 cm in its long axis terminating immediately proximally to the level of the olecranon and with involvement of the underlying medulla (Figure 2). Chest CT scan and whole body bone scan confirmed no evidence of disease elsewhere. A core needle biopsy was performed which showed features consistent with a parosteal osteosarcoma.

**Figure 3:**
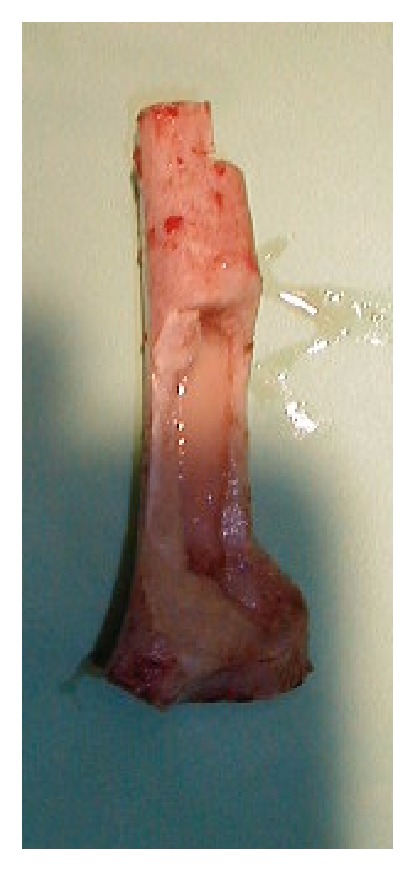
The irradiated bone segment.

**Figure 4:**
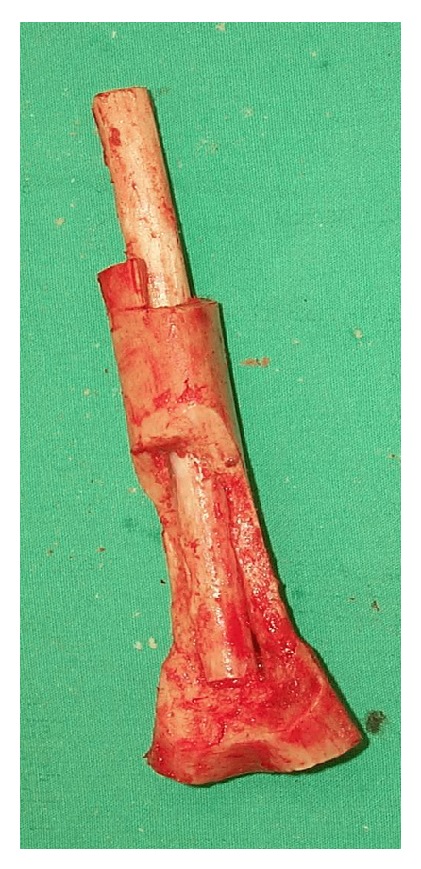
Fibula graft inside the irradiated bone segment.

**Figure 5:**
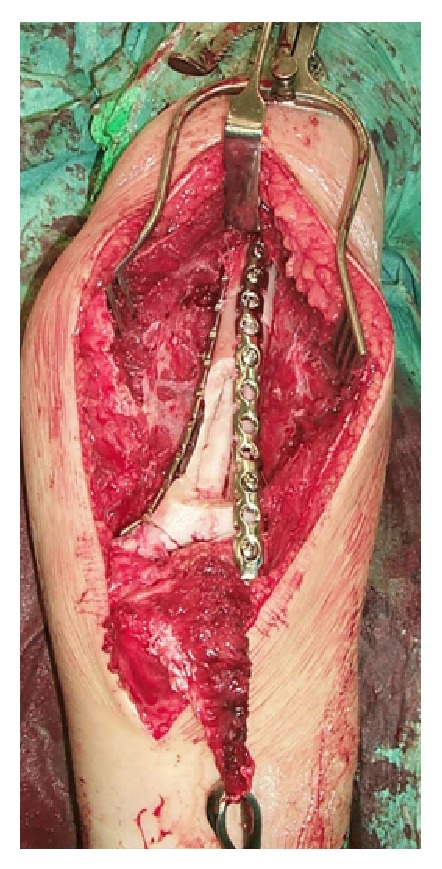
The construct was held in place with 2 plates running up each side of the humerus.

**Figure 6:**
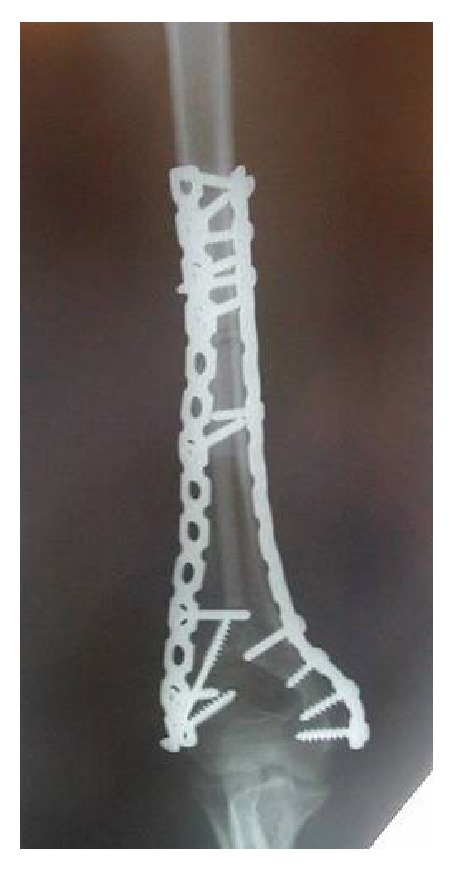
Postoperative X-ray.

Reconstructing a segmental defect at this site is fraught with difficulties. Although we have reported good results with distal humerus replacements [7], we anticipated long term complications such as aseptic loosening using this option in a 12-year-old. Reconstruction with bone transport was not attractive because of the length of time that would have been required and the associated risk of elbow stiffness. Reconstruction with strut allografts requires an adequate bone banking service and presents inherent risks of graft rejection and imperfect fit of the donated graft to the recipient bone defect. Therefore we planned to do an en bloc resection of tumor bearing bone, extracorporeal irradiation of excised bone segment to eradicate the tumoral cells, and reimplantation of the autograft with preservation of the elbow joint. We resected 10 cm of the humerus, stopping 2 cm from the elbow joint and dividing the bone a further 10 cm proximal to this. We then stripped the tumor from the bone surface and underlying intramedullary canal using osteotomes, curettes, and a burr. Samples were sent for histology and microbiology. The humerus segment was wrapped in a sterile moist swab immersed in saline containing 2 grams of vancomycin and placed in a sterile bag. This was placed in another sterile bag and was securely packed in a sterile container. This container was taken to the radiotherapy suite, where the segment bone was irradiated with 90 Gy, and the container was returned to the operation theater. Transport and irradiation took about one hour, during which time the host bone was prepared for reconstruction. The irradiated humeral segment was reimplanted, inserting a nonvascularised fibular autograft through the middle of it to provide extra stability (Figures 3 and 4). The construct was held in place with 2 plates running up each side of the humerus (Figures 5 and 6).

**Figure 7:**
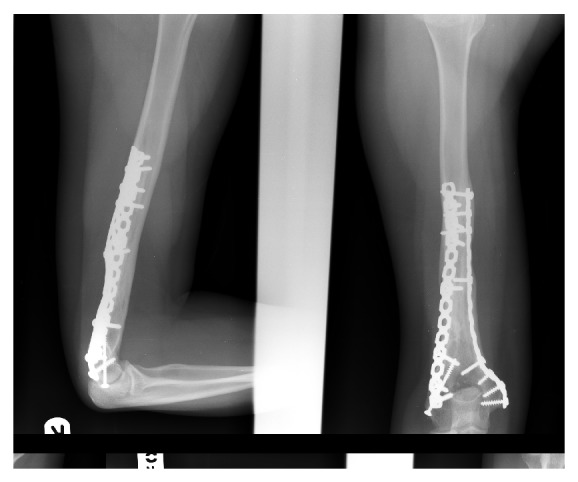
Lateral and anteroposterior X-ray 10 years after surgery. These show full healing and consolidation of the graft with excellent union at both ends of the graft.

The patient started mobilization exercises of the elbow from the third postoperative day. On the fifth postoperative day, the day of his discharge from our hospital, the range of motion of his elbow was 0–90 degrees. Six weeks after surgery, she had flexion of 0–120 degrees. About 4 months after surgery, she regained full flexion and extension of the elbow and full pronation and supination of the forearm. Elbow X-ray showed excellent union at both ends of the graft. At last follow-up ten years after surgery, she continues to have excellent function with full range of movement and can play sports without problems. X-ray of the right humerus showed full healing and consolidation of the graft (Figure 7). The patient had no recurrence of the tumour.

## 3. Discussion

Parosteal osteosarcoma is a rare form of low-grade malignant osteosarcoma, representing approximately 4% of all osteosarcoma [8, 9]. It is a slow-growing neoplasm that originates from the surface of the cortex and forms a bony mass in the soft tissue. It mainly affects the female. The most frequent location is the posterior aspect of the distal femur (in 60% of cases), the proximal humerus, and the proximal tibia. Medullary bone is rarely involved, except in cases with prior treatment or a long history of growth [10].

Histologically, the lesions are frequently well differentiated, with a low propensity to metastasize, and the prognosis is better compared with conventional osteosarcoma [11].

The differential diagnosis may include different entities such as myositis ossificans, ossifying hematoma, osteochondroma, extraosseous osteosarcoma, desmoplastic fibroma, and osteoma.

In the past the preferred treatment of osteosarcoma parosteal, as in other types of osteosarcoma, was amputation [12]. Today, the amputation has been replaced in many cases by limb salvage, with the en bloc resection of the tumor and reconstruction by prostheses, allograft, autograft, or combined methods. In order to reduce the risk of local recurrence, it is important to perform wide-margin procedures [13]. Marginal or intralesional excision was associated with a very high rate of local recurrence [12].

In the case we described, we opted for an “en bloc” excision, extracorporeal irradiation, and reimplantation, because the patient was young and with good prognosis. We excluded the use of a tumor prosthesis because being a young patient it would have been exposed to greater wear and to an increased risk of complications such as breakage, infections, aseptic loosening of prosthesis with the risk of repeated procedures, and finally even amputation. The positioning of an intercalary endoprosthesis was not possible for the short distance between the distal resection and the joint line.

Even the allograft is not free from complications, such as the risk of disease transmission, the high rate of infection, and nonunion and pathological fractures.

The irradiated autograft solves many of these problems. Surely, the primary advantage of irradiated reimplants is to have a precise fit being the patient's own bone. In this way we avoid all the problems related to the adaptation of the shape and size.

Extracorporeal irradiation and reimplantation of autogenous tumor bone was first described in 1968 by Spira and Lubin [14]. Since then, various authors have used this method and have shown the validity of this. In the cases reported in the literature, the dose of radiation varies between 50 and 300 Gy [1, 14–16]. Although a large dose of radiation can be applied to the resected autograft because there is no risk of radiation injury to the surrounding soft tissue, many authors have suggested that higher doses are not necessary for sterilisation of the tumor [1]. Voggenreiter et al. have shown that doses of radiation used for sterilization do not destroy the bone induction properties of bone matrix. In scanning electron microscopy, they found no deleterious effects of irradiation on the surface structure of cortical bone and the extracorporeal irradiation of cortical bone with oncologically effective doses of 1 and 5 kGy did not substantially impair the integration of the grafts [17].

Local recurrence has been rarely reported in literature after extracorporeal irradiation. These recurrences occurred in the surrounding soft tissues, most likely attributable to inadequate resection [2, 18].

In our case we have not had any complication, but local complications are associated with this technique such as nonunion, pathological fracture, infection, and bone necrosis. It is not clear how much the chemotherapy contributes to these complications in these patients [15]. However, the complication rate is not greater than that related to other procedures, while functional results are very satisfactory in most cases [19].

Another complication of using this procedure may be damage to the articular cartilage resulting after irradiation.

In case of osteoarticular graft, Uyttendaele et al. observed disintegration of articular cartilage and resorption of the subchondral bone with this type of reconstruction. Therefore, in ostearticular procedure they prefer a hybrid reconstruction that combines an irradiated autograft with a tumor joint arthroplasty [2]. However, a histologic study performed by Hatano et al. shows the presence of viable chondrocytes cells on specimens previously treated with irradiation with a dose of 60 Gy [20].

Studies show the mature joint cartilage is radio resistant to high-dose therapeutic irradiation [20, 21].

Because the irradiation causes a reduction in mechanical properties of bone, in order to reduce the risk of nonunion it is important to create a stable system through long term support of the metalwork, such as plates, screws, and nails. In our case, the distal resection being 2 cm from the joint line, we could not use a nail and so we used a fibular autograft. We believe that in some cases a fibular graft may be a good additional help in fixing and we can take advantage of the osteoconductive and osteogenic properties of this bone to speed up the union.

Extracorporeal irradiation and reimplantation is a valid and inexpensive technique for the treatment of bone tumors when there is reasonable residual bone stock.

We believe that this technique may provide a rewarding alternative to allografting and in some cases can replace the use of tumor implants with excellent functional and oncological results.
